# The accuracy of fully automated algorithms for surveillance of healthcare-onset *Clostridioides difficile* infections in hospitalized patients

**DOI:** 10.1017/ash.2022.32

**Published:** 2022-03-16

**Authors:** Suzanne Desirée van der Werff, Mikael Fritzing, Hideyuki Tanushi, Aron Henriksson, Hercules Dalianis, Anders Ternhag, Anna Färnert, Pontus Nauclér

**Affiliations:** 1 Division of Infectious Diseases, Department of Medicine Solna, Karolinska Institutet, Stockholm, Sweden; 2 Department of Infectious Diseases, Karolinska University Hospital, Stockholm, Sweden; 3 Uppsala University Hospital, Uppsala, Sweden; 4 Department of Data Processing & Analysis, Karolinska University Hospital, Stockholm, Sweden; 5 Department of Computer and Systems Sciences, Stockholm University, Stockholm, Sweden

## Abstract

We developed and validated a set of fully automated surveillance algorithms for healthcare-onset CDI using electronic health records. In a validation data set of 750 manually annotated admissions, the algorithm based on *International Classification of Disease, Tenth Revision* (ICD-10) code A04.7 had insufficient sensitivity. Algorithms based on microbiological test results with or without addition of symptoms performed well.

*Clostridioides difficile* infections (CDI) pose a problem within healthcare worldwide. In Europe, nearly 190,000 patients are hospitalized with CDI every year.^1^ The most dominant risk factor for CDI is previous treatment with antibiotics, but CDI outbreaks also occur in healthcare institutions.^2^ Effective surveillance is important to register adverse events, to enable quick response to outbreaks, and to evaluate control measures. However, most surveillance is based on time-consuming and resource-intensive manual review of patient records, which is also prone to subjective interpretation and surveillance bias.^3^ In this study, we developed and evaluated the performance of a set of fully automated rule-based surveillance algorithms, including free-text analysis, for healthcare-onset (HO) CDI in hospitalized patients using electronic health record (EHR) data.

## Methods

In this observational study, we used prospectively entered EHR data from the Karolinska University Hospital which is stored in a research infrastructure called Health Bank–Swedish Health Record Research Bank at DSV/Stockholm University, as previously described.^4,5^ The study was approved by the Regional Ethical Review Board in Stockholm under permission no. 2016/2309-32 and 2012/1838-31/3.

**Fig. 1.**
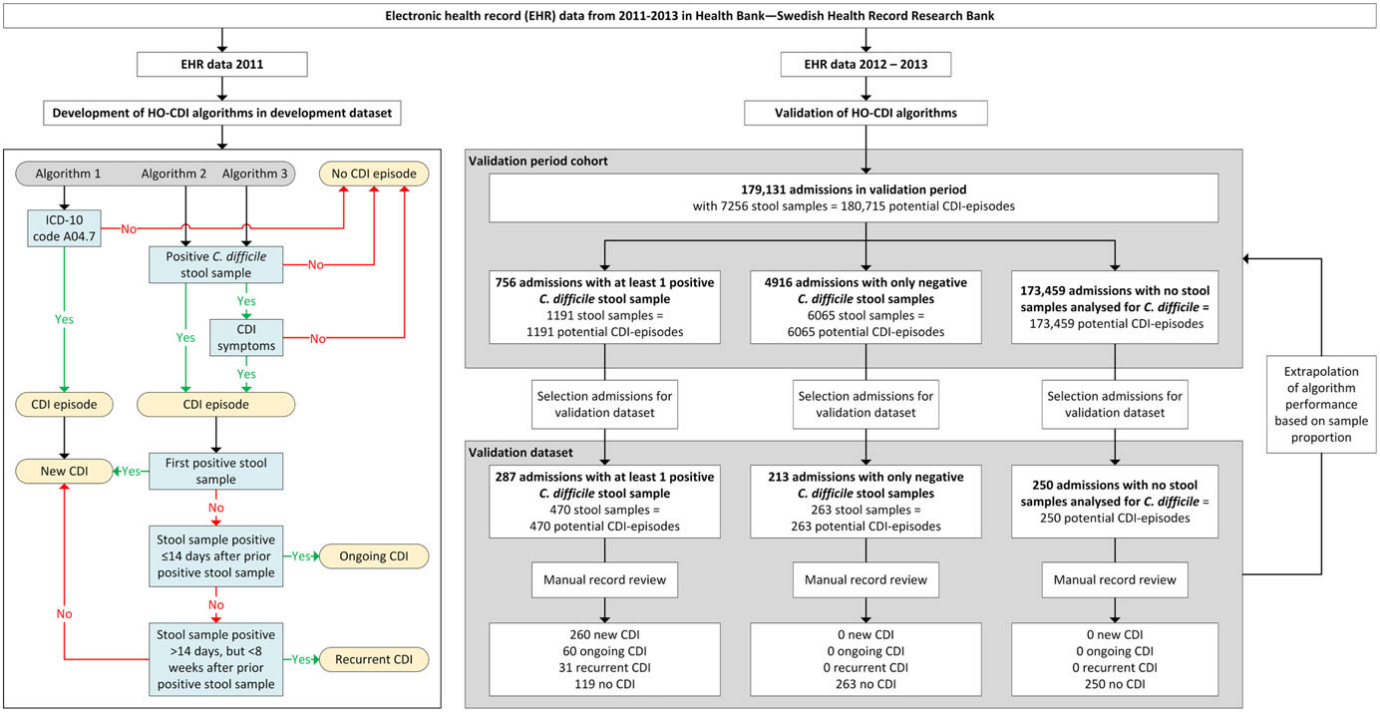
Flow chart of study and flow diagram of the 3 rule-based surveillance algorithms for HO *Clostridioides difficile* infection. Note. CDI, *Clostridioides difficile* infection; HO, healthcare-onset. CDI episode: All stool samples analyzed for *C. difficile* during admission were regarded as potential CDI episodes, and each admission without stool samples analyzed for *C. difficile* counted as 1 potential CDI episode.

We developed and assessed 3 rule-based algorithms to detect HO-CDI, CDI detected any time during hospital admission, according to the definition of the European Centre for Disease Prevention and Control (ECDC) (Fig. 1)^6^: (1) algorithm 1: ICD-10 code A04.7 (enterocolitis due to *C. difficile*) during admission; (2) algorithm 2: positive stool sample with *C. difficile* toxin or toxin-producing *C. difficile* present during admission; and (3) algorithm 3: algorithm 2 and CDI symptoms.

All stool samples analyzed for *C. difficile* during admission were regarded as potential CDI episodes, and each admission without stool samples analyzed for *C. difficile* counted as 1 potential CDI episode. Stool samples were defined as positive when *C. difficile* toxin A and/or B or a toxin-producing *C. difficile* organism was detected (based on cell cytotoxicity assay and toxigenic culture). For the presence of CDI symptoms, the 7-day infection window period (IWP) of the Centers for Disease Control and Prevention (CDC) was used^7^, that is, symptoms had to be present within 3 days before or after the day of the positive stool sample.

The development data set consisted of admissions with a positive stool sample for *C. difficile* between January 2011 and December 2011 (n = 561). CDI symptoms in all free-text medical notes ±3 days around (n = 14,107) all positive stool samples (n = 682) were manually annotated to find possible ways of describing diarrheal stool and pseudomembranous colitis. Based on a list of key words detected from these descriptions, we created a regular expression (regex) for scanning free-texts in the EHR. This regex also incorporated a negation cue to discard texts saying, for example, ‘no diarrhea.’ CDI episodes were defined as ongoing if a positive stool sample result occurred within 14 days after a previous positive stool sample, and was defined as recurrent if a positive stool sample result occurred >14 days but <8 weeks after a previous positive stool sample.^6,7^ Other CDI episodes were regarded as new infections.

The performance of the algorithms was assessed in a validation data set of 750 randomly selected admissions during 2012 and 2013. The validation data set was created by sampling 3 groups: (1) admissions with at least 1 positive stool sample for *C. difficile* (n = 287 of 756); (2) admissions with only negative stool samples for *C. difficile* (n = 213 of 4,916); and (3) admissions with no stool samples analyzed for *C. difficile* (n = 250 of 173,459). The 750 admissions were manually annotated via medical record review to determine whether patients fulfilled the ECDC CDI definition. These 750 admissions added up to 983 potential CDI episodes (within the 3 groups 470, 263 and 250 possible CDI-episodes, respectively) (Fig. 1).

Algorithm performance was evaluated by assessing the sensitivity, specificity, positive predictive value (PPV), and negative predictive value (NPV). To obtain performance estimates of the algorithms in the total hospital population, estimates were extrapolated to all patients hospitalized during 2012–2013 based on the sampling proportions within the 3 groups described above.^5^ The confidence interval (CI) for the extrapolated estimates were calculated as the 2.5th and 97.5th percentiles of point estimates obtained from 10,000 bootstrap samples for each of the 3 groups. To account for uncertainty, the bootstrapping was performed before extrapolating the proportions from the validation dataset to the validation period cohort. Analyses were performed using R version 3.6.1 software (R Foundation for Statistical Computing, Vienna, Austria).

## Results

The 750 hospital admissions within the validation cohort comprised 719 patients (641 adults and 78 children), of whom 253 (35.2%) had a CDI. Moreover, 225 (35.1%) of 641 adults had a CDI, and 28 (35.9%) of 78 children had a CDI. Compared to patients without CDI, patients with a CDI were older (median age, 69 vs 58 y), had a longer length of stay (median days, 17 vs 5), had a higher Charlson comorbidity index (median, 2 vs 1), and had a higher in-hospital mortality rate [9.5%, (24 of 253) versus 1.9% (9 of 466); all *P* < .001].

According to the manual record review of the 983 potential CDI episodes present within the 750 admissions 351 were confirmed CDI episodes: 260 (26.4%) were new, 60 (6.1%) were ongoing, and 31 (3.2%) were recurrent (Fig. 1). For the algorithm performance only the new CDI episodes were used. Algorithm 1, based on ICD-10 code A04.7, had a sensitivity of 0.442 (95% CI, 0.381–0.504) in correctly identifying CDI episodes (Table 1). Algorithms 2 and 3 had sensitivities of 1.000 (95% CI, 0.999–1.000) and 0.992 (95% CI, 0.980–1.000), respectively, and both had a specificity of 1.000 (95% CI, 0.999–1.000). Using algorithm 2, 12 patients in the validation set were misclassified as positive compared to 6 patients using algorithm 3, and algorithm 3 misclassified 2 patients as negative (for details see footnote Table 1).

**Table 1.** Performance Characteristics of 3 Rule-Based Algorithms for Classifying Healthcare-Onset Clostridioides *difficile* Infection According to the ECDC Definition

Algorithm	Among 983 Potential CDI Episodes in 750 Admissions in the Validation Data Set, No.				Extrapolated Results to All Hospitalized Patients During the Validation Period (CDI-Episodes, N = 180,715)				
	True Positives	False Positives	False Negatives	True Negatives	Sensitivity (95% CI)	Specificity (95% CI)	PPV (95% CI)	NPV (95% CI)	AUC (95% CI)
Algorithm 1^a^	115	69	145	654	0.442 (0.381–0.504)	0.999 (0.998–0.999)	0.531 (0.397–0.682)	0.998 (0.998–0.998)	0.720 (0.701–0.739)
Algorithm 2^b^	260	12	0	711	1.000 (0.999–1.000)	1.000 (0.999–1.000)	0.956 (0.932–0.978)	1.000 (0.999–1.000)	0.999 (0.999–1.000)
Algorithm 3^c^	258	6	2	717	0.992 (0.980–1.000)	1.000 (0.999–1.000)	0.978 (0.958–0.992)	1.000 (0.999–1.000)	0.996 (0.993–1.000)

Note. ECDC, European Center for Disease Control; AUC, area under the receiver operating characteristic (ROC) curve; NPV, negative predictive value; PPV, positive predictive value. CDI-episode: all stool samples analyzed for *C. difficile* during admission were regarded as potential CDI-episodes, and each admission without stool samples analyzed for *C. difficile* counted as one potential CDI-episode. The extrapolated results of the algorithms from the validation data set to the validation period cohort (2012–2013) were based on the sampling proportion of potential CDI episodes from the 3 different groups: (1) admissions with a positive stool sample for *C. difficile*; (2) admissions with only negative stool samples for *C. difficile* and; (5) admissions without stool samples analyzed for *C. difficile*.

a Algorithm 1: ICD-10 code A04.7.

b Algorithm 2: Positive stool sample with *C. difficile* toxin or toxin-producing *C. difficile*: 12 false-positive cases due to having positive stool sample, but no symptoms (n = 8) and misclassification of CDI by annotator being true-positive cases (n = 4).

c Algorithm 3: Positive stool sample with *C. difficile* toxin or toxin-producing *C. difficile* and CDI symptoms: 6 false positives due to negation rule for symptoms not working (n = 1), wrong detection of symptom by regex (n = 2) and misclassification of CDI by annotator being true-positive cases (n = 3); 2 false-negative cases due to symptoms missed by algorithm (n = 1) and misclassification of CDI by annotator being true-negative case (n = 1). Algorithm 1 could only differentiate between (new) CDI or no CDI and classified 184 (18.7%) as CDI episodes. Algorithms 2 and 3 classified, 272 (27.7%) and 264 (26.9%) as new CDI episodes, 61 (6.2%) and 60 (6.1%) as ongoing CDI episodes, and 33 (3.4%) and 32 (3.3%) as recurrent CDI episodes, respectively. The results in the table are based only on new CDI episodes.

## Discussion

Fully automated algorithms based on microbiological data with or without free-text analysis of symptoms performed well for surveillance purposes in detecting CDI, whereas an algorithm based on ICD-10 code had insufficient sensitivity. This inadequacy was related to poor recording of the CDI ICD-10 code despite positive stool tests and symptoms for CDI. The algorithm based on only microbiological tests tended to slightly overestimate the prevalence of CDI, and the algorithm that also included analyses of free text slightly reduced false positives. However, this improvement came at the expense of being computationally more challenging and would also require adaptation to local EHR systems. The small difference between the algorithms based on microbiological data only compared to the addition of free-text analysis of symptoms corresponded to the situation in which testing for *C. difficile* was primarily performed when patient exhibited symptoms. In our hospital, *C. difficile* testing was initiated only based on the clinical decision of the physician, and all stool samples sent to the microbiology laboratory for this purpose where analyzed (no rejection criteria based on stool consistency or frequency of testing, for example). However, in settings with more liberal use of *C. difficile* microbiological testing, free-text analysis might show greater benefit because it could reduce false-positive results.

Only 3 studies have shown the benefit of fully automated surveillance for CDI bases on microbiology data.^8–10^ However, 2 studies did not validate their results against record review via ECDC or CDC guidelines.^8,9^ The remaining study validated their algorithm against record review according to CDC guidelines.^10^ The specificity of their algorithm for HO-CDI, based on a positive stool sample, was similar to our 2 algorithms using positive stool samples with or without CDI symptoms. The sensitivities of both our algorithms were higher than their sensitivity. However, this difference was caused by the relatively poor performance in one of their participating hospitals compared to the other 3 hospitals in their study.

The strengths of our study included the extensive availability of EHR data, which enabled us to simulate the performance of epidemiological surveillance using real-world, real-time data, and the application of the same testing guidelines and diagnostic methods for CDI throughout the entire study period. Our study also had limitations. We used data from only 1 hospital over a limited period, so the applicability of the algorithms to other acute-care settings, especially in regard to different testing strategies over time and across institutions, may limit the generalizability of our results. For example, during the study period, immunoassays (EIAs) or nucleic acid amplification tests (NAATs) were not used as diagnostic methods, which reduced the risk of overdiagnosis in our study. However, the introduction of NAAT might lead to the overdiagnosis of CDI, both by algorithms and manual annotation, which should be considered when implementing this algorithm during later periods or at other institutions.

In conclusion, algorithms based on microbiological test results only are likely to perform well in hospitals with symptom indications for *C. difficile* testing, while free-text analysis of medical notes could improve surveillance algorithm performance if more liberal indications for *C. difficile* testing are used.
